# Do ambiguous images provide psychological insights? Testing a popular claim

**DOI:** 10.7717/peerj.19022

**Published:** 2025-02-20

**Authors:** Richard Wiseman, Caroline Watt

**Affiliations:** 1 University of Hertfordshire, Hatfield, Hertfordshire, United Kingdom; 2 University of Edinburgh, Edinburgh, Scotland, United Kingdom

**Keywords:** Psychology, Perception, Illusion, Ambiguous, Personality, Thinking style

## Abstract

Social media posts and websites claim that the way in which people perceive ambiguous images reveals insights into their personality and thinking style. To explore this notion, participants indicated the first image that they perceived in four ambiguous pictures (Duck-Rabbit, Younger-Older Woman, Rubin’s Vase and Horse-Seal), and completed a Five Factor personality measure along with scales relating to optimism, procrastination, holistic thinking, and decision-making style. Many of the claims received no empirical support and so constitute a new type of psychological myth. Future research could explore why these claims remain popular with the public and why people frequently share the material with others. In addition, several significant and interesting findings emerged, including associations between Duck-Rabbit, personality, and optimism, and Younger-Older Woman and age. Possible future research into these phenomena is discussed.

## Introduction

Recent popular social media posts and website pages have claimed that the way in which people first perceive ambiguous images (*i.e.,* pictures that can be interpreted in different ways) reveals insights into their personality and thinking style. In one example, people are shown an image that can be seen as either a young woman or man, and informed that seeing the woman first suggests that they are optimistic and curious, whereas perceiving the man first indicates that they are honest and tend to avoid impulsive decisions (New Zealand Herald, 2016). Regardless of their veracity, these claims have the potential to make a valuable contribution to research.

If the claims are untrue, then they would constitute a new form of psychological myth (*i.e.,* phenomena that are popular with the public but lack scientific support). Researchers have previously identified several such myths, including the notion that people only use 10% of their brains, that eye movements are a reliable indicator of lying, that human memory acts like a video recorder, and that people’s star signs dictate the course of their lives (*e.g.*, Lilienfeld et al., 2010; Wiseman et al., 2012; Swami et al., 2015; Hupp & Wiseman, 2023). New myths are valuable because they highlight novel misunderstandings in the public perception of psychology, and help to extend existing work into the formation and maintenance of such beliefs (*e.g.*, Standing & Huber, 2003; Cho, 2022; Redifer & Jackola, 2024; Rodríguez-Prada, Orgaz & Cubillas, 2022).

Alternatively, if the claims are valid, they have the potential to contribute to research examining the perception of ambiguous images (for reviews, see: Long & Toppino, 2004; Rodríguez-Martínez & Castillo-Parra, 2018). This is especially likely because much of the previous work in the area revolves around the speed at which people switch between different interpretations of pictures (*e.g.*, Bergum & Bergum, 1979; Christman, Sontam & Jasper, 2009; Kanai, Bahrami & Rees, 2010; Wiseman et al., 2011). For example, Blake & Palmisano (2021) examined the relationship between creativity and the perception of ambiguous images. Participants rated the degree of switching experienced whilst looking at the Necker Cube and the Spinning Dancer, and the results revealed that the former was related to participants’ scores on a measure of divergent thinking. In later work, Koivisto & Pallaris (2024) showed that cognitive flexibility played a key role in this effect. In contrast to this work, the claims currently being made on social media and websites focus on the possible correlates of people interpreting the ambiguous images in certain ways. To date, researchers have carried out a relatively small amount of research into this topic.

For example, McManus et al. (2010) reported archive data from a 1953 BBC project examining whether handedness, age and sex affected the perception of the well-known Duck-Rabbit image (seen as either a rabbit looking to the right or a duck looking to the left; Jastrow, 1899). Nearly 4,000 members of the public completed a postcard containing their answers to several questions (including those relating to which hand participants tended to use for various activities) along with a description of how they perceived the image. Several patterns emerged in the data, including men being more likely than women to see the image as a duck, older participants being more likely to perceive the figure as a rabbit, and handedness not being related to the perception of the image.

Brugger & Brugger (1993) showed that the way in which people interpret an ambiguous image could be influenced by the cultural context in which the picture was perceived. Both children and adults in Switzerland were shown the Duck-Rabbit image around Easter or in October. Presumably primed by the traditional Western association between Easter and bunnies, participants tended to perceive the image as being a rabbit around Easter and a duck in October. In similar work, Kihlstrom et al. (2018) presented Australian and American participants with the Arizona Whale-Kangaroo (which can look like an outline of a whale or a kangaroo). As predicted, compared to the American participants, the Australian participants were more likely to see the kangaroo.

Balcetis & Dunning (2006) explored the role of motivation by rewarding participants for seeing an ambiguous image in a certain way. For example, in one study, participants were rewarded for spotting images of either farm animals or sea creatures. When shown the Horse-Seal image (seen as either a horse or a seal; Fisher, 1968), those motivated to spot farm animals were more likely to perceive the image as a horse, whereas those being rewarded for spotting sea creatures were more likely to see it as a seal.

Several studies have explored whether social anxiety impacts on the perception of ambiguous films in which actors wearing ‘light suits’ (outfits on which points of lights are placed on major joints) can be perceived as either walking towards or away from the observer (for reviews, see Van De Cruys, Schouten & Wagemans, 2013; Peng et al., 2021). This work explores the notion that anxious participants are motivated to perceive the ambiguous stimulus in a way that reduced the sense of threat (*i.e.,* the figures as walking away from them) and has obtained mixed results.

Wiseman et al. (2011) conducted two studies examining whether participants’ creativity (measured with both a self-rated scale and a standard creativity test) was related to the number of times they switched their perception of the Duck-Rabbit image. During both studies participants also described the animal that they first saw when viewing the image. No significant relationships emerged between participants’ level of creativity and whether they first saw the image as a rabbit or a duck.

Nicholls, Churches & Loetscher (2018) examined the relationship between participants’ age and their perception of the Younger-Older Woman image (seen as either a younger woman facing away from the observer or as an older woman facing towards them; Boring, 1930). Participants were shown the image for 500 milliseconds and then asked to estimate the age of the woman. The study showed that the estimates provided by older participants (aged over 30) were significantly higher than those of younger participants (those aged 18 to 30). Brouwer et al. (2021) repeated the experiment using a similar procedure and obtained the same results. In addition, Brouwer et al. (2021) examined whether participants’ age affected which interpretation of the image they saw first and failed to find any effect.

In short, a small amount of previous research has examined the potential correlates associated with perceiving ambiguous images in certain ways. However, to our knowledge, none of this work has explored the claims currently found on many social media posts and websites. The current study addresses this issue and centres upon four images that frequently feature in psychological research into ambiguous pictures and often appear in these claims, namely: the Duck-Rabbit; Younger-Older Woman; Horse-Seal; and Rubin’s Vase (seen as either two faces in profile or a vase; Rubin, 1915). Participants indicated the first image that they perceived in these four pictures, and then completed a series of measures relating to personality, optimism, procrastination, holistic thinking, and decision-making style.

The study examined whether the interpretation of these images is associated with personality/thinking style in general, and tested the following hypotheses (based on claims being made on some social media posts and websites):

(i) Duck-Rabbit: Seeing the duck first is associated with lower levels of emotional stability and optimism, whereas seeing the rabbit first is associated with higher levels of procrastination (*e.g.*, Tran, 2023a);

(ii) Rubin’s Vase: Seeing the faces first is associated with higher levels of detail-oriented thinking, whereas seeing the vase first is associated with higher levels of spontaneous decision making and lower levels of detail-oriented thinking (Times of India, 2022);

(iii) Younger-Older Woman: Seeing the older woman first is associated with higher levels of agreeableness and logical decision making, whereas seeing the younger woman first is associated with higher levels of independent decision making (Tran, 2023b);

(iv) Horse–Seal image: Seeing the seal first is associated with higher levels of detail-oriented holistic thinking and analytical decision making (Hayes, 2022).

## Materials & Methods

### Ambiguous images

Duck-Rabbit: This first appeared in an 1892 German engraving by an unknown artist and was popularised by psychologist Jastrow (1899).

Rubin’s Vase: This appeared in a late nineteenth century American postcard (Seckel, 2004) and is more commonly associated with psychologist Rubin (Rubin, 1915).

Younger-Older Woman: This appeared as an anonymous German 1880 postcard, before being adapted by American cartoonist William Ely Hill in 1915 and popularised by psychologist Boring (Boring, 1930).

Horse-Seal: This image was created by Fisher (1968) as a new stimulus to be used in research on ambiguous images.

Copies of all four images are available from the authors upon request.

The authors have permission to use the following instruments from the copyright holders.

### Ten-item personality inventory (TIPI; Gosling, Rentfrow & Swann Jr, 2003)

This 10-item inventory measures the major traits in the Five Factor model (Extraversion, Agreeableness, Conscientiousness, Emotional Stability, Openness to Experiences). For each item, participants rate the degree to which two adjectives (*e.g.*, ‘Extraverted, Enthusiastic’) describe them on a 7-point Likert scale from 1 (Strongly disagree) to 7 (Strongly agree). Higher scores indicate higher levels of Extraversion, Agreeableness, Conscientiousness, Emotional Stability, and Openness.

### Holistic cognition scale (HCS; Lux, Grover & Teo, 2021)

This 12-item scale measures four aspects of holistic/analytic cognition: Attention (focusing on parts *versus* the whole); Causality (focusing on internal dispositions/motivations *versus* context /circumstances); Contradiction (focusing on propositions being true/false *versus* being open to contradiction) and Change (seeing change as linear and incremental *versus* constant and cyclical). Participants rate the degree to which each statement (*e.g.*, ‘Where you put an ornament is just as important as the ornament itself’) describes them on a 7-point Likert scale from 1 (Completely disagree) to 7 (Completely Agree). Higher scores are related to more holistic cognitive tendencies across the four domains and lower scores reflect more analytic cognitive tendencies.

### The Optimism–pessimism short scale–2 (SOP2; Nießen et al., 2022)

This 2-item scale provides a general measure of optimism. Participants first rate the degree to which a general statement outlining the nature of optimism describes them on a 7-point Likert scale from 1 (Not at all optimistic) to 7 (Very optimistic). The same approach is then used for a second item on pessimism. Higher scores indicate greater levels of optimism.

### General procrastination scale (GPS-9; Sirois, Yang & Van Eerde, 2019)

This 9-item scale provides a general measure of procrastination. Participants rate the degree to which each statement (*e.g.*, ‘In preparing for some deadlines, I often waste time by doing other things’) describes them on a 5-point Likert scale from 1 (Strongly disagree) to 5 (Strongly agree). Higher scores indicate greater levels of procrastination.

### General Decision-Making Styles (GDMS; Scott & Bruce, 1995)

This 25-item scale measures five decision-making styles: Rational (logical evaluation of alternatives), Intuitive (a reliance on hunches and feelings), Dependent (advice and direction from others), Avoidant (postponing and avoiding decisions), and Spontaneous (a desire to make decisions as soon as possible). Participants rate the degree to which each statement (*e.g.*, ‘I plan my important decisions carefully’) describes them on a 5-point Likert scale from 1 (Strongly disagree) to 5 (Strongly agree). Due to time constraints, the Avoidant subscale was not used in the study. Higher scores indicate higher levels of Rational, Intuitive, Dependent and Spontaneous decision making.

### Participants

Participants (*n* = 300, mean age = 39.06 years; *SD* = 11.90; range 18–79. Age categories: 18–30, *n* = 83; 31–40, *n* = 92; 41–50, *n* = 72; 51–60, *n* = 38; Over 60, *n* = 15) were recruited from the crowdsourcing platform Prolific Academic (https://www.prolific.com/). Several studies have validated the use of this type of platform within psychological research (*e.g.*, Crump, McDonnell & Gureckis, 2013; Enochson & Culbertson, 2015). Estimating a prior expected effect size wasn’t possible due to the small amount of past research in the area, however, the predetermined sample size had a good chance of detecting a medium sized effect (*d* = 0.5, *p* < 0.05, 2-tailed, power = 0.9).

### Procedure

The study was approved by the University of Hertfordshire Health, Science, Engineering & Technology Ethics Committee (number LMS/SF/UH/05799) and run in September 2024. Participants were recruited using Prolific Academic and the study was presented *via* the Qualtrics platform (https://www.qualtrics.com/). After reading an information sheet and providing written consent, participants were asked to enter their year of birth and age (this acted as a data validity check as their reported age could be checked against their year of birth). They were shown the Duck-Rabbit image and told: “Please take a look at this image. This picture can be viewed in different ways. What did you see when you first looked at the image?” (Options: Duck; Rabbit; Neither or something else). The same wording was used for the Rubin’s Vase (Options: Vase; Faces in profile; Neither or something else) and Younger-Older Woman (Options: Younger Woman looking away from you; Older Woman looking towards you; Neither or something else), and the Horse-Seal (Options: Horse or Donkey; Seal: Neither or something else). Participants then completed the TIPI, HCS, SOP2, GPS-9 and GDMS. All participants viewed the images and completed the questionnaires in the same order. They were then shown a debrief sheet and thanked for participating. Participants were given a small financial reward for their time.

## Results

All participants, conditions, measures, and data were reported and included in the analyses. The number of participants reporting each interpretation of the ambiguous images were as follows: Duck-Rabbit (Duck = 213; Rabbit = 85; Neither = 2); Younger-Older Woman (Younger = 226; Older = 69; Neither = 5); Rubin’s Vase (Vase = 180; Faces = 103; Neither = 7); Horse-Seal (Horse=275; Seal = 21; Neither = 4). Participants indicating ‘Neither or other’ were excluded from analyses, and point bi-serial correlations were used to compare the scores obtained from the two groups with each of the measures. All analyses are reported in Table 1. The mean questionnaire ratings and standard deviations associated with each of the statistically significant relationships are reported in Table 2.

**Table 1:** Point bi-serial correlations (rpb) and associated 2-t *p*-values (in parentheses) between participants’ perception of each ambiguous image and their scores on the TIPI, SOP2, GPS-9, age, HCS, and GDMS measures.

	**Image**			
**Measure**	**Duck-** **Rabbit**	**Rubin’s** **vase**	**Younger- Older woman**	**Horse-** **Seal**
TIPI	**.13**	.01	−.06	.10
Extraversion	**(.02)**	(.87)	(.30)	(.09)
TIPI	.02	−.08	.02	−.03
Agreeableness	(.69)	(.18)	(.74)	(.63)
TIPI	**.11**	−.08	.10	.02
Conscientiousness	**(.05)**	(.17)	(.11)	(.78)
TIPI	**.12**	−.11	.08	.04
Emotional stability	**(.04)**	(.07)	(.17)	(.45)
TIPI	.05	**.17**	−.03	−.05
Openness	(.35)	**(.003)**	(.64)	(.40)
SOP2	**.16**	−.01	−.02	.02
Optimism	**(.007)**	(.83)	(.71)	(.70)
GPS9	−.08	.03	−.07	.01
Procrastination	(.18)	(.63)	(.22)	(.81)
Age	.003	−.006	**.13**	−.02
	(.97)	(.91)	**(.03)**	(.75)
HCS	.02	−.11	−.02	−.01
Attention	(.68)	(.07)	(.70)	(.86)
HCS	.09	−.02	−.003	.08
Causality	(.13)	(.69)	(.95)	(.16)
HCS	.09	.07	−.004	.03
Contradiction	(.13)	(.24)	(.95)	(.67)
HCS	.03	−.05	.03	−.02
Change	(.64)	(.45)	(.61)	(.71)
GDMS	−.04	.00	.09	−.03
Rational	(.53)	(.99)	(.12)	(.61)
GDMS	.05	−.08	−.03	**.17**
Intuitive	(.40)	(.18)	(.63)	**(.004)**
GDMS	−.007	.01	**−.12**	−.01
Dependent	(.90)	(.85)	**(.04)**	(.82)
GDMS	.10	.06	**−.16**	**.14**
Spontaneous	(.07)	(.30)	** (.006)**	** (.01)**

**Notes.**

Significant correlations (*p* < .05) highlighted in bold.

Duck *n* = 213, Rabbit *n* = 85; Vase *n* = 180, Faces *n* = 103; Younger woman *n* = 226, Older woman *n* = 69; Horse *n* = 275, Seal *n* = 21.

**Table 2:** The images, measures, means, and standard deviations associated with each of the statistically significant relationships reported in Table 1.

**Image**	**Measure**	**Outcome**
Duck-Rabbit	TIPI Extraversion	Duck *M* = 3.34, *SD*= 1.53 Rabbit *M* = 3.81, *SD*= 1.65
	TIPI Conscientiousness	Duck *M* = 5.04, *SD*= 1.40 Rabbit *M* = 5.39, *SD*= 1.27
	TIPI Emotional Stability	Duck *M* = 4.28, *SD*= 1.44 Rabbit *M* = 4.65, *SD*= 1.39
	SOP2	Duck *M* = 4.20, *SD*= 1.32 Rabbit *M* = 4.65, *SD*= 1.30
Rubin’s Vase	TIPI Openness	Faces *M* = 5.10, *SD*= 1.11 Vase *M* = 4.65, *SD*= 1.25
Younger-Older woman	GDMS Spontaneous	Younger *M* = 2.60, *SD*= .86 Older *M* = 2.27, *SD*= .80
	GDMS Dependence	Younger *M* = 3.33, *SD*= .92 Older *M* = 3.06, *SD*= .96
	Age	Younger *M* = 38.26, *SD*= 11.53 Older *M* = 41.80, *SD*= 12.66
Horse-Seal	GDMS Intuitive	Horse *M* = 3.45, *SD*= .75 Seal *M* = 3.94, *SD*= .74
	GDMS Spontaneous	Horse *M* = 2.48, *SD*= .82 Seal *M* = 2.95, *SD*= 1.20

The following findings are based on the significant relationships that are identified in Tables 1 and 2.

**Duck-Rabbit:** As hypothesised, seeing the duck first was associated with lower levels of emotional stability and optimism. However, there was no support for the hypothesis that seeing the rabbit first was associated with procrastination. In addition, seeing the rabbit first was associated with higher scores on TIPI Extraversion and Conscientiousness.

**Rubin’s Vase:** Contrary to the hypotheses, seeing the faces first was not associated with detail-oriented thinking, and seeing the vase first was not associated with spontaneous decision making and detail-oriented thinking. However, perceiving the faces first was associated with higher scores on TIPI Openness.

**Younger-Older woman:** Contrary to the hypotheses, seeing the older woman first was not associated with agreeableness and logical decision making, and seeing the younger woman first was associated with lower (not higher) levels of independent decision making. However, perceiving the younger woman first was associated with higher scores on GDMS Spontaneous.

The perception of the Younger-Older Woman image was associated with participants’ age, with those seeing the older woman first being older than those seeing the younger woman first. Previous work (Nicholls, Churches & Loetscher, 2018; Brouwer et al., 2021) has suggested younger participants (18–30 years old) provide lower estimates the age of the woman than older participants (Over 30). The current study didn’t aim to replicate this work and didn’t ask participants to estimate the age of the woman. However, Brouwer et al. also examined whether participants’ age (18–30 *vs* Over 30) was related to which interpretation of the image they saw first, and failed to find an effect. For completeness, we carried out a similar Chi-squared analysis (with Yates’ continuity correction) between participants’ age (18–30, Over 30) and the interpretation of the images (Young, Old), and obtained a non-significant result (*χ*^2^ = 1.64, *df* = 1, *N* = 295, *p* = .26).

**Horse-Seal:** Contrary to the hypotheses, seeing the seal first was not associated with detail-oriented thinking and analytical decision making. However, seeing the seal first was associated with higher scores on GDMS Intuitive and GDMS Spontaneous.

## Discussion

This study examined whether the way in which people perceive ambiguous images is related to their personality and thinking style. Many of the hypotheses (based on claims being made in social media posts and on websites) were not substantiated. This included the notion that people who see the rabbit first in the Duck-Rabbit image tend to procrastinate more than those who see the duck first, those who perceive the older woman first in the Younger-Older Woman image are more agreeable than those who see the younger woman first, and those who see the faces first in Rubin’s Vase are more detail oriented than those who see the vase first. In addition, it’s claimed that those seeing the younger woman first in the Younger-Older Woman image are more independent than those who see the older woman first, whereas the data showed the opposite relationship. The association between the perception of Duck-Rabbit, optimism and emotional stability supported the hypotheses. However, the effect sizes are very small and so may have little/no real impact on peoples’ everyday thinking or behaviour. Nevertheless, they might be of theoretical value and could be the topic of future work aimed at identifying whether such effects are genuine and, if so, investigating the underpinning mechanisms.

Given the lack of empirical support for these claims, it is interesting to consider the mechanisms that may explain their popularity. For example, research suggests that people often have little insight into the nature of their personality (*e.g.*, Vazire & Mehl, 2008; Vazire, 2010) and abilities (*e.g.*, Zell & Krizan, 2014), and thus might struggle to know whether the descriptions provided by social media posts and websites are accurate. Second, research into alleged paranormal divination (*e.g.*, astrology, graphology, psychic readings, and palmistry) suggests that people tend to be impressed by statements about their personality that are general and positive (*e.g.*, Hyman, 1977; Dickson & Kelly, 1985; Dean & Kelly, 2003; O’Keeffe & Wiseman, 2005). In line with this work, many of the alleged insights from the perception of ambiguous images lack specificity and usually frame the observer in a positive light. Third, confirmation bias may also play a key role in the process, with people remembering the instances wherein the alleged predictions matched their personality and thinking, and forgetting the times that this was not the case (White, Brockett & Overstreet, 1993). Future work in this area could build on these ideas, further exploring the mechanisms that underpin the formation and maintenance of belief in these claims.

The popularity of these social media posts and websites may also prove interesting to researchers seeking to understand internet memes. Previous work suggests that these messages and images are often shared because they help to reduce stress, to build a sense of identity and community, and to promote positive emotions (for a review, see Adiga & Padmakumar, 2024). Future work could examine whether the success of the types of social media posts and websites discussed here involve these factors or help to yield novel insights into shareability. For example, the success of the posts seems to depend, at least to some extent, on promoting a sense of debate that more closely mirrors arguments surrounding the blue-gold dress (for a review see; González Martín-Moro et al., 2018).

Some of the additional statistically significant findings (*e.g.*, the perception of Rubin’s Vase and openness, the Horse-Seal and intuitive decision making, and the Duck-Rabbit and extraversion and conscientiousness) appear somewhat isolated, are not related to previous research, or claims being made in social media posts and websites. As such, they may be the result of multiple analyses. In addition, spontaneous decision making was significantly associated with the way in which participants perceived both the Younger-Older Woman and Horse-Seal images. However, existing research into spontaneous decision-making offers few clues about a potential mechanism, with most of the work linking the style with a range of negative outcomes, including incompetency (Parker, Bruine De Bruin & Fischhoff, 2007), and low agreeableness and low conscientiousness (El Othman et al., 2020). Future research could investigate whether this relationship is genuine and, if so, explore possible mechanisms. Finally, older participants tended to see the older woman first whereas younger participants tended to see the younger woman first. However, again this effect is small but could be of theoretical importance and thus the topic of future research.

In terms of study strengths, the stimuli were presented in the same media as those in the original claim (*i.e., via* the web), involved the same response options, and used the same instructional set (*i.e.,* participants were asked to indicate which image they saw first). However, in terms of limitations, the personality and thinking style measures were self-report, and future work could examine whether the pattern of findings replicate with more task-based measures. In addition, the study employed participants drawn from a general pool, and additional work could involve individuals who are especially likely to engage with, and to share, material relating to the claims under examination. Finally, the order of the response options was not randomised. This was in line with the way in which the stimuli and options are presented on social media and websites, and thus made for a more realistic test of the hypotheses. However, future research could randomise these options to minimise and examine any potential ordering effects.

## Conclusions

In sum, this paper examined recent popular claims, made in social media posts and on websites, that the way in which people interpret ambiguous images provides an insight into their personality and thinking style. Many of the findings did not support these claims and so these claims constitute a new form of psychological myth. Future research could examine why these claims are popular despite being inaccurate. However, it’s important not to throw the baby out with the bath water, with two sets of interesting findings emerging in the data. First, some of the associations between the Duck-Rabbit image and participants’ personality and level of optimism were significant. Second, in line with previous research, there was a significant association between participants’ age and their interpretation of the Younger-Older Woman image. Future research could attempt to replicate these findings and investigate potential underlying mechanisms. It is hoped that this research has laid the foundations for additional work into these curious phenomena and will motivate other researchers to perceive ambiguous images in a new and informative way.

## Supplemental Information

10.7717/peerj.19022/supp-1 Supplemental Information 1 Raw data from the study, showing all of the measures

## Additional Information and Declarations

### Data Availability

The following information was supplied regarding data availability: The raw measurements are available in the Supplemental File.
